# Influence of Polyvinylpyrrolidone on Thermoelectric Properties of Melt-Mixed Polymer/Carbon Nanotube Composites

**DOI:** 10.3390/mi14010181

**Published:** 2023-01-11

**Authors:** Beate Krause, Sarah Imhoff, Brigitte Voit, Petra Pötschke

**Affiliations:** 1 Leibniz-Institut für Polymerforschung Dresden e.V. (IPF), Hohe Str. 6, 01069 Dresden, Germany; 2 Chair Organic Chemistry of Polymers, Technische Universität Dresden, 01062 Dresden, Germany

**Keywords:** thermoelectric, polymer composites, nanotubes, electron doping

## Abstract

For thermoelectric applications, both p- and n-type semi-conductive materials are combined. In melt-mixed composites based on thermoplastic polymers and carbon nanotubes, usually the p-type with a positive Seebeck coefficient (S) is present. One way to produce composites with a negative Seebeck coefficient is to add further additives. In the present study, for the first time, the combination of single-walled carbon nanotubes (SWCNTs) with polyvinylpyrrolidone (PVP) in melt-mixed composites is investigated. Polycarbonate (PC), poly(butylene terephthalate) (PBT), and poly(ether ether ketone) (PEEK) filled with SWCNTs and PVP were melt-mixed in small scales and thermoelectric properties of compression moulded plates were studied. It could be shown that a switch in the S-value from positive to negative values was only possible for PC composites. The addition of 5 wt% PVP shifted the S-value from 37.8 µV/K to −31.5 µV/K (2 wt% SWCNT). For PBT as a matrix, a decrease in the Seebeck coefficient from 59.4 µV/K to 8.0 µV/K (8 wt% PVP, 2 wt% SWCNT) could be found. In PEEK-based composites, the S-value increased slightly with the PVP content from 48.0 µV/K up to 54.3 µV/K (3 wt% PVP, 1 wt% SWCNT). In addition, the long-term stability of the composites was studied. Unfortunately, the achieved properties were not stable over a storage time of 6 or 18 months. Thus, in summary, PVP is not suitable for producing long-term stable, melt-mixed n-type SWCNT composites.

## 1. Introduction

Thermoelectrics (TE) is becoming increasingly important in the search for environmentally friendly energy conversion. Here, thermoelectric voltage is generated with suitable materials through a temperature gradient on both sides of the material harvesting, e.g., waste heat [1]. The Seebeck coefficient S, which is calculated from the generated thermoelectric voltage (U) divided by the applied temperature difference (ΔT), is used to characterize the TE properties. A second parameter is the power factor PF which is calculated from the product of the electrical volume conductivity σ and the squared Seebeck coefficient (PF = S^2^·σ) [2,3]. In addition, if values of thermal conductivity are available, also the figure of merit ZT can be calculated as ZT = PF·*T*·*κ*^−1^, with T as the absolute temperature and κ as thermal conductivity.

To improve the environmental aspect of this technology, the aim of the present work is to replace the commonly used TE materials which quite often contain rare earth metals with more environmentally friendly materials. Rare earth metals are partly toxic, expensive, associated with geopolitical risks during extraction, and difficult to process. When polymers are applied as substitutes for TE materials, in addition to intrinsically conductive polymers (ICPs), electrically conductive polymer composites (CPCs) are an alternative. It is particularly advantageous to use thermoplastic matrices that can be processed by melt processing methods and high aspect ratio carbon nanotubes (CNTs) are used as fillers to form the electrically conductive network at low concentrations, even if the achieved PF and ZT values are still much lower than for traditional materials. For the fabrication of a TE module, the combination of p- and n-type materials is favorable, and they are then arranged alternately. If commercial CNTs, which typically exhibit p-type behavior, are incorporated into polymers, the composites usually also show the p-type. This could be shown for polypropylene (PP) [4,5,6], polycarbonate (PC) [7,8,9,10], poly(ether ether ketone) (PEEK) [10,11], polyvinylidene fluoride (PVDF) [9,12], and poly(butylene terephthalate) (PBT) [9] based melt-mixed composites. The achievement of n-type behavior is more challenging. Recently, it could be shown that the incorporation of p-type SWCNTs can lead to n-type composites when using specific polymer matrices, such as acrylonitrile butadiene styrene (ABS) [9] or different kinds of polyamides [9,13]. As further alternatives to produce n-type composites, n-type CNTs can be incorporated, such as nitrogen-doped CNTs [14], or p-type CNTs can be combined with additives such as ionic liquids [15], polyethylenimine (PEI) [16], or polyethylene glycol (PEG) [5,17,18] before being added to the polymer matrix. In this context, the stability of the n-type character and the negative Seebeck coefficients over the storage time of the composite is a crucial point, which unfortunately has not been studied sufficiently and reported in the literature. For example, Lu et al. found that, for the approach to melt-mix PEG and SWCNTs in PP, even after 18 months of storage in air, the n-type behavior of the composites was preserved with only a slight change in the Seebeck coefficient from −56.6 to −32.6 µV/K. However, in a study by Yu et al. [19], the n-type character of PEI-doped CNT buckypapers was lost already after 18 days of storage (change from −34 µV/K to 10 µV/K).

The evaluation of TE modules based on thermoplastic polymer composites was for example reported by Hewitt et al. with p- and n-type layers based on PVDF/CNT prepared by solution drop-casting [16,20] and by Luo et al. for melt-mixed PP/SWCNT composites without and with PEG addition [5].

The additive for the polymer/CNT composite that is investigated in depth in this study is polyvinylpyrrolidone (PVP), which is commonly reported as a non-toxic n-type additive for SWCNTs. This polymeric dopant was described by Piao et al. [21] by treating SWCNT buckypapers with PVP dissolved in N-methyl-2-pyrrolidone (NMP). It was found that the electrical conductivity of SWCNT buckypapers was reduced by PVP addition from around 12.5 × 10^−3^ to 2.5 × 10^−3^ S/m, and the Seebeck coefficient changed from around 40 µV/K to over −30 µV/K. The authors explain this switching from p- to n-type with the nitrogen heteroatoms in the pyrrolidin-2-on side group of PVP, which are supposed to act as potential electron donor materials [21]. Hata et al. [22] studied the dispersions of SWCNTs in a PVP-NMP solution drop cast on a polyimide sheet as an n-type thermoelectric materials. A negative Seebeck coefficient of −46 µV/K was obtained for the PVP-SWCNT composite layer in comparison to 32.9 µV/K of the untreated SWCNT layer. In this study, the volume conductivity of the layers increased with the PVP addition from around 300 S/m up to 1200 S/m. In a different work, Ryan et al. [23] described an all-organic textile thermoelectric module consisting of n-type poly(ethylene terephthalate) (PET) yarns coated by dripping an aqueous MWCNT-PVP (ratio 1:4) nanocomposite dispersion down the vertically hanging yarns. The individual MWCNT-PVP n-type yarns presented a Seebeck coefficient of −14 µV/K with a conductivity of around 100 S/m and a PF of 10^−2^ µW/(m·K²). Nonoguchi et al. [24] conducted a screening experiment with a wide range of additives including PVP. They prepared dopant–dimethyl sulfoxide (DMSO) solutions and poured these on SWCNTs followed by homogenization with an Ultra Turrax and formed buckypapers by filtration. The results from this work using PVP as n-type dopant show a promising Seebeck coefficient value of around −55 µV/K in comparison to an S-value of the undoped SWCNT buckypapers of +49 µV/K.

PVP was also reported as a successful n-type additive for non-oxidised graphene flakes (NOGF) by Novak et al. [25,26]. Thereby, the graphene flakes were exfoliated by bath sonication using a PVP-DMSO solution followed by vacuum filtration. The Seebeck coefficient of the PVP-NOGF material was measured to be −45.5 µV/K and the conductivity was about 301,000 S/m (PF = 621 µW/(m·K^2^)) at 313 K. The use of graphene as a carbon substrate for PVP was also reported by Cho et al. [27] who studied multilayer thin films composed of alternated PEI-stabilized double-walled carbon nanotubes and PVP-stabilized graphene nanocomposites. The thin films were prepared from aqueous nanofiller dispersions containing PEI or PVP using ultrasonic the treatment and dipping the poly(ethylene terephthalate) substrates into these dispersions. The overall 80-bilayer DWNT-PEI/graphene-PVP thin film presented a high conductivity of 30,000 S/m and a Seebeck coefficient of −80 µV/K (PF = 190 µW/(m·K^2^)).

From the studies published in the literature, it can be concluded that PVP can act as an n-dopant for CNTs, despite different results. It can therefore be expected that the use of PVP in melt-mixed polymer/CNT composites will result in a negative Seebeck coefficient. It should be highlighted that, for the first time, PVP is to be used as an n-dopant in melt-mixed composites. In the present study, it was first determined whether PVP acts as an n-dopant for the SWCNT material applied in our study using SWCNT buckypapers. PVP was then incorporated as an additive in various concentrations into polymer/SWCNT composites based on three different polymer matrices, namely polycarbonate (PC), poly(butylene terephthalate) (PBT), and poly(ether ether ketone) (PEEK). The TE properties such as the Seebeck coefficient and power factor of those melt-mixed composites were examined. In addition, the long-term stability of the TE properties after storage under ambient conditions was investigated.

## 2. Materials and Methods

### 2.1. Materials

Three different kinds of commercially available polymers were selected for this study. Polycarbonate (PC) Makrolon 2600 granules (Covestro AG, Leverkusen, Germany), poly(ether ether ketone) (PEEK), Vestakeep 1000P powder (Evonik Industries AG, Essen, Germany), and poly(butylene terephthalate) (PBT) Ultradur B4500 granules (BASF SE, Ludwigshafen, Germany) were used.

The carbon nanotubes selected for this study were SWCNT Tuball^TM^ (OCSIAl, Luxembourg) with a carbon content of 75% [28,29]. The selection was based on a former study comparing different kinds of CNTs [9]. RAMAN spectra of this CNT type are published in the supporting information of [10]. Thermoelectric parameters of the SWCNT powder were described in [9]. This kind of SWCNTs is abbreviated as Tuball.

**Figure 1:**
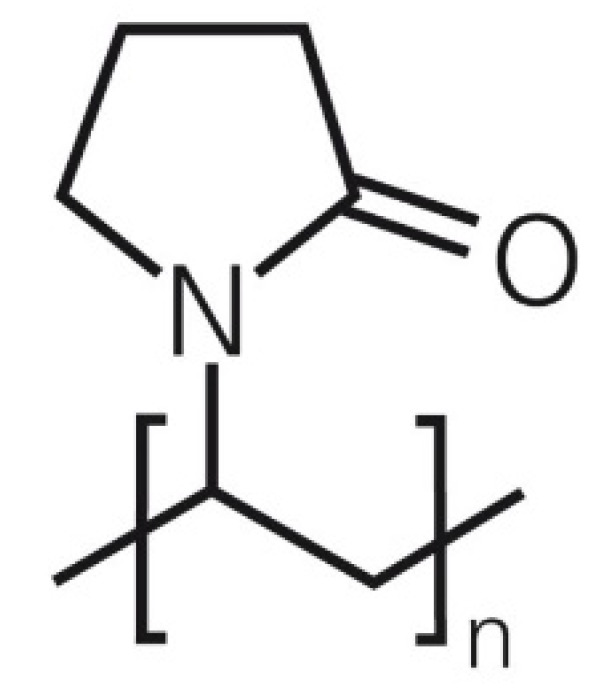
Structure of poly(vinylpyrrolidone) (PVP).

As additive polyvinylpyrrolidone (PVP, K30, Sigma-Aldrich, CAS 9003-39-8, St. Louis, MO, USA) was used (Figure 1). PVP is an amorphous, water-soluble polymer with an average molecular weight of 40.000 g/mol.

### 2.2. Methods

The buckypaper preparation was optimized and performed as follows: 22.5 mg of SWCNTs were dispersed in 30 mL ethanol (VWR chemicals, Mw 46.07 g/mol, CAS 64-17-5). The dispersion was obtained using ultrasonication with UP400St (Hielscher Ultrasonics, Teltow, Germany) using a 3 mm diameter sonotrode H3 (cycle 100%, amplitude 20%) for 10 (2 times 5 min) minutes without any cooling. The dispersion was filtered using a PTFE filter (diameter of 47 mm, pore size 0.2 μm; Munktell & Filtrak GmbH, Bärenstein, Germany) and a water pump. Pristine SWCNT buckypapers were obtained upon drying in air at room temperature.

For the preparation of PVP containing buckypaper, the SWCNT dispersion was prepared as described above but only 5 (or 6) minutes of ultrasonic treatment was applied. Afterward, 2 g of a 10 wt.% PVP solution in ethanol was added to the SWCNT dispersion and 5 (or 6) minutes of ultrasonic treatment was again applied without cooling. The dispersion was filtered using a PTFE filter and a water pump and dried in air at room temperature.

The composites were prepared using a conical twin-screw micro-compounder Xplore 15 (Xplore, Sittard, The Netherlands) with a volume of 15 cm^3^. The fillers (SWCNTs, PVP) were pre-mixed by shaking in a glass. The SWCNT/PVP premix was filled into the microcompounder alternately with the polymer granules. After cooling down, the obtained extruded strands were compression molded into plates (60 mm diameter, 0.5 mm thickness) using the hot press PW40EH. The preparation conditions for melt compounding and compression molding are described in Krause et al. [18].

The thermoelectric (TE) characterization was carried out in a measuring device developed and constructed at IPF Dresden [30]. The measuring temperature was set to 40 °C with four or eight temperature variations up to ±8 K. More details are given in Krause et al. [18]. The measurements were performed within two weeks after sample preparation.

Thermogravimetric analysis (TGA) was performed on PVP granules by using a Q 5000 analyzer (TA Instruments, Hüllhorst, Germany) in air and nitrogen atmosphere. The heating rate was 10 K/min, and a temperature range from 25 °C up to 800 °C was applied.

The morphological characterization of the composites was performed using scanning electron microscopy (SEM) by means of a Carl Zeiss Ultra plus microscope combined with an SE2 detector. Before imaging, the composite strands were cryo-fractured in liquid nitrogen, and the surfaces were coated with 3 nm platinum.

## 3. Results

### 3.1. Thermal Degradation of PVP

PVP, used as the additive for thermoelectric composites, is very hygroscopic [31], has good adhesive properties (e.g., in glue sticks and metal glue), and is used, for example, as a binder in tablets. It is an amorphous polymer with a glass transition temperature (T_g_) between 110 and 180 °C depending on the molecular weight [32]. For the grade K30 used in our study, a T_g_ of 162 °C was reported [32].

We first studied its thermal stability by means of TGA, especially at the processing temperatures of PC, PBT, and PEEK. The test in nitrogen is intended to simulate that the additive is encapsulated in the polymer melt shortly after it is filled in, thus practically eliminating its contact with air.

**Figure 2:**
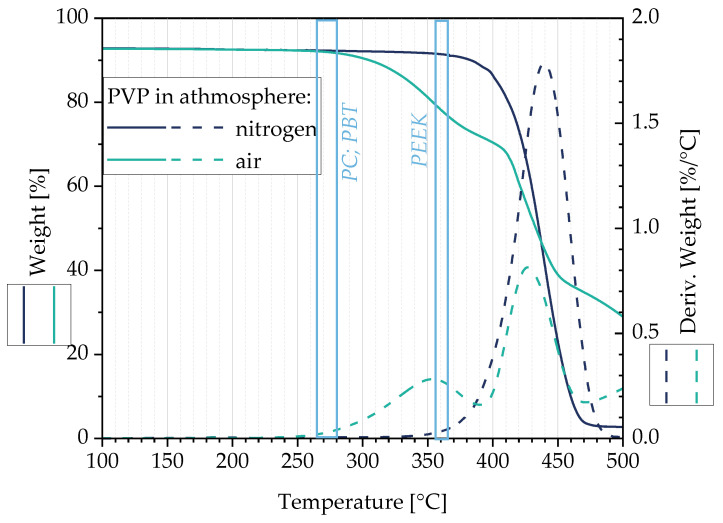
Thermal degradation behavior of PVP.

Figure 2 shows that the thermal decomposition in a nitrogen atmosphere starts at 340 °C, which is higher than the temperature used for composite preparation using PC and PBT. However, when producing PEEK composites at 360 °C, PVP degradation has already started. The main degradation takes place at 440 °C.

In air, a two-step degradation behaviour of PVP is observed. The degradation starts at 270 °C with maxima at 350 °C and at 430 °C. Assuming that air is rapidly excluded during melt processing, minimal molecular degradation can be expected when producing the PBT (265 °C) and PC-based composites (280 °C). When producing the PEEK composites (360 °C), significant degradation of the PVP has to be expected.

Loría-Bastarrachea et al. [33] found that during the thermal degradation of PVP under nitrogen, vinylpyrrolidone is the main volatile product, which means that the predominant mechanism during the thermal degradation of this polymer is the depolymerisation of the polymeric backbone to the monomer. In addition, simultaneous reactions take place in which oligomers were formed. In air, the oxidation of PVP leads to carbon dioxide and pyrrolidone around 300–400 °C [34]. The degradation of PVP starts with the breakdown of the side groups [35] with corresponding double bond formation. Bogatyrev et al. [36] described the appearance of pyrrolidone and vinyl pyrrolidone ions in mass spectra after thermal degradation.

### 3.2. Single-Walled Carbon Nanotube Buckypaper with PVP

In the next step, the n-doping effect of PVP on the SWCNTs used in the present study was investigated. Since each SWCNT type has a slightly different molecular structure, which is influenced by, e.g., the defect density, a varying n-doping effect of the PVP can be expected. In addition, the sample preparation also plays a role in the achievable properties.

To study the PVP effect, SWCNT buckypapers were produced without and with PVP by applying a total of 10 min of ultrasonic treatment. For the pure SWCNT buckypaper, a volume conductivity of 12,545 ± 270 S/m, a Seebeck coefficient of 62.2 ± 0.6 µV/K, and a power factor of 48.6 µW/(m·K^2^) were determined. After PVP addition, the volume conductivity increased to 14,631 ± 288 S/m and the Seebeck coefficient decreased to 33.0 ± 1.7 µV/K. Thus, the power factor decreased to 16.0 µW/(m·K^2^). When the duration of ultrasound treatment was increased to two times 6 min, an S-value of −8.1 ± 2.0 µV/K could be measured.

The result shows that PVP is reducing the S-value for the SWCNTs used here; although, it was strongly dependent on the preparation conditions of the buckypaper. A negative Seebeck coefficient as reported in the literature [21,22,23,24,25] was achieved. With this result, it must of course also be taken into account that other types of CNTs were used in the above-mentioned studies, which differ in their thermoelectric properties. It is assumed that the explanation given by Piao et al. [21] that the nitrogen heteroatom in the pyrrolidine-2-on side group of PVP (as seen in Figure 1) acts as an electron donor to the SWCNTs can be applied here.

### 3.3. Polycarbonate-Based Composites

**Figure 3:**
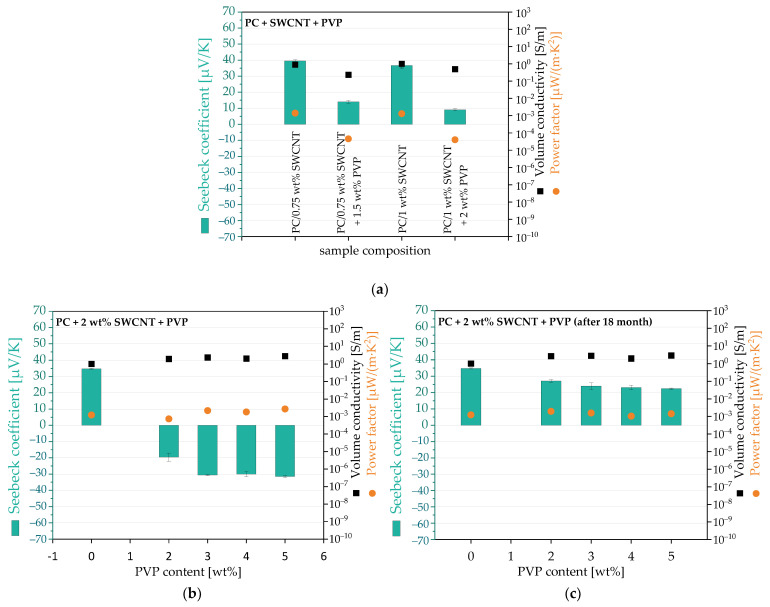
TE properties of PC composites with SWCNT Tuball: SWNT content of 0.75 and 1 wt% and PVP in the ratio 1:2 (**a**), PC/2 wt% SWCNT Tuball composites with different contents of PVP, first measurements (**b**), and measurements repeated after 18-month storage under ambient conditions (**c**).

The PVP was added to PC/Tuball composites in different amounts. The thermoelectric results are summarized in Figure 3 and Table S1. For the composites with 0.75 and 1 wt.% Tuball, the addition of PVP (ratio SWCNT:PVP = 1:2) only led to a reduction of the Seebeck coefficient but still kept positive values (Figure 3a). This is an indication that PVP is able to reduce the Seebeck coefficient also in melt-mixed PC composites. The volume conductivity is only slightly reduced after PVP addition. Due to the decreased Seebeck coefficient, the PF values were two decades lower than in the composites without PVP.

At 2 wt.% Tuball, with PVP addition, negative Seebeck coefficient values were achieved. With 3–5 wt.% PVP, S-values around −30 µV/K could be measured (Figure 3b). The volume conductivity was not affected by the PVP incorporation. The PF values were around 0.002 µW/(m·K^2^). It seems that in combination with a certain PVP content, the SWCNT content had to reach a certain threshold concentration to achieve the switching to negative S-values.

After a storage time of 18 months under ambient laboratory conditions, the thermoelectric measurements were repeated at the same specimens without any additional preparation steps. The aim was to investigate the long-term stability of the thermoelectric properties of these composites. As shown in (Figure 3c), a positive S-value has now been measured for all composites with PVP. The Seebeck coefficients were still slightly less positive than for the composite without PVP, but the n-doping effect has been lost. This means that PVP cannot be used to produce a long-term stable composite with a negative Seebeck coefficient. Unfortunately, there are no further studies in the literature that have investigated such a time-dependent change, e.g., on PVP-doped pure SWCNT samples. As the conductivity of the composites did not change during the 18 months of storage, it is reasonable to conclude that the PVP itself is unstable.

**Figure 4:**
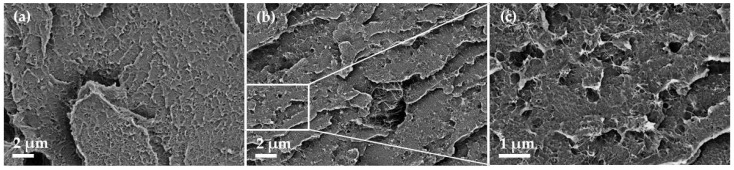
SEM image of PC composites with 2 wt% SWCNT (**a**) and PC with 2 wt% SWCNT + 5 wt% PVP (**b**,**c**).

The morphological characterisation of the PC composites was performed using SEM (Figure 4). For both composites without or with PVP, a homogeneous SWCNT dispersion is visible. If PVP has been incorporated then small holes (smaller than 500 µm) can be seen in the composite surface (Figure 4b,c). It is assumed that this is due to the PVP. This means that the PVP particles are quite finely dispersed in the PC matrix and not miscible with the PC.

### 3.4. PBT-Based Composites

The PVP addition to PBT/2 wt.% Tuball composites was performed to further verify the approach. The thermoelectric measurements show that the S-values decreased with increasing PVP content. At 8 wt.% PVP addition, there was no change of the sign, but only a decrease in S-values from 59.4 µV/K to 7.9 µV/K (Figure 4, Table S2). This reduction confirms that PVP can influence the conduction type also in the PBT matrix in the direction of n-type. However, negative Seebeck coefficients could not be reached. Since the S-value increases again at 10 wt.% PVP addition, an even higher PVP addition is unlikely to result in a negative Seebeck coefficient for the composite.

Additionally, for these composites, measurements were repeated on the same sample specimens after 6 months (Figure 4b). As with the PC composites, an increase in S-values was observed after storage. At around 48–52 μV/K, the S values were slightly lower than for the composite without PVP. This means that no thermoelectrically long-term stable composites can be produced for PBT either. The electrical conductivities were constant over the storage time. Thus, there was no change in the conductive network of the SWCNTs. It can be therefore concluded again that the change in thermoelectric properties has to be attributed to changes in PVP.

**Figure 5:**
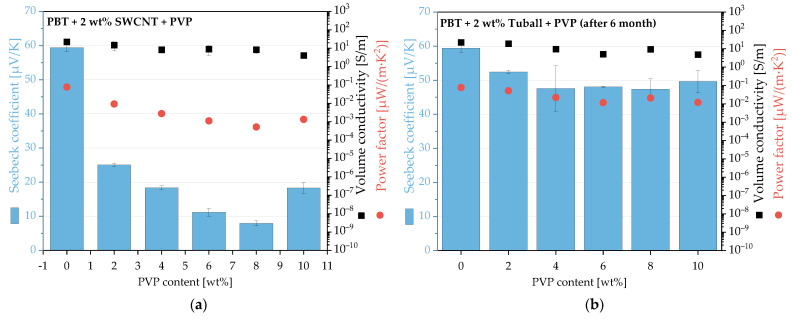
TE properties of PBT composites with 2 wt% SWCNT Tuball and different contents of PVP, first measurements (**a**), and measurement repeated after 6 months (**b**).

**Figure 6:**
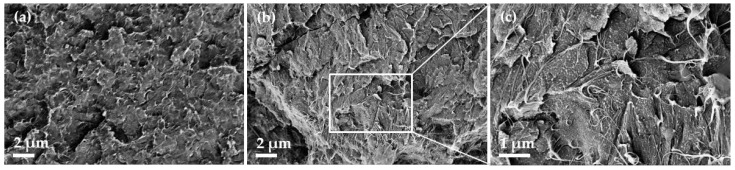
SEM image of PBT composites with 2 wt% SWCNT (**a**) [18] and PBT with 2 wt% SWCNT + 10 wt% PVP (**b**,**c**).

Morphological characterization of the PBT composites by SEM showed homogeneous SWCNT dispersion in the polymer matrix both without and with PVP (Figure 5). The CNTs can be recognised as long lines. When PVP is added, very small dot-shaped humps can be seen at the polymer surface at the highest magnification (Figure 6c). These could be PVP particles that are also homogeneous dispersed.

### 3.5. PEEK-Based Composites

The third polymer used as a matrix was PEEK. In contrast to PC and PBT composites, the addition of PVP leads to slightly higher Seebeck coefficients (Figure 5, Table S3). Thus, the S-value increased from 48.0 µV/K (without PVP) to 54.3 µV/K (@ 3 wt% PVP). The volume conductivity was also slightly higher after the PVP addition and increased marginally from 6 S/m (without PVP) to approx. 13 S/m (with PVP).

When the thermoelectric measurements were repeated after 17 months, only slightly changed values were measured. The S-values were slightly higher after storage. However, since the error ranges of these measurements were relatively high, sometimes 6–12%, it cannot be judged as a significant deviation. The volume conductivities were constant over the storage period.

One reason for the hardly measurable effect of PVP addition in PEEK composites could be the high processing temperature of 360 °C. The TGA results (Section 3.1) had shown that the degradation of PVP to monomers has already started, especially when the PVP is still in contact with air at the beginning of the melt mixing. It was assumed that with molecular degradation, a possible n-doping effect is also reduced.

**Figure 7:**
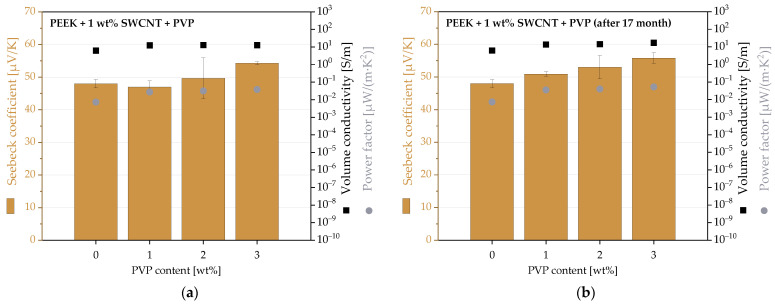
TE properties of PEEK composites with 1 wt% SWCNT Tuball and different contents of PVP, first measurements (**a**), and measurement repeated after 17 months (**b**).

**Figure 8:**
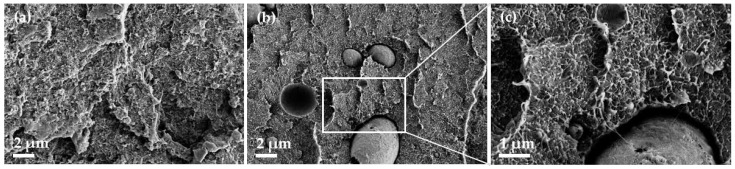
SEM image of PEEK composites with 1 wt% SWCNT (**a**) and PEEK with 1 wt% SWCNT + 3 wt% PVP (**b**,**c**).

Morphological characterization of the PEEK composites by SEM showed homogeneous SWCNT dispersion in the polymer matrix both with and without PVP (Figure 7). Noticeable are the 2–5 µm cavities and spheres that appear when PVP has been added (Figure 8b,c). It can be concluded that these are PVP particles and that PVP is not miscible with PEEK. The worst PVP dispersion could be one reason why PVP is not an n-dopant for SWCNTs in PEEK. The large particle size of PVP could reduce the contact between SWCNTs and PVP in the composite even if SWCNTs and PVP were incorporated as a premix during melt mixing.

## 4. Discussion

As reported in the literature, PVP was able to change the conduction behavior of the here-used SWCNTs from p- to n-type, whereby this result was strongly dependent on the preparation condition of the doped buckypaper. However, an n-doping effect can be seen as the Seebeck values decreased after PVP addition in the ethanol-SWCNT dispersion from 62.2 µV/K to 33 µV/K (10 min ultrasonication) or −8 µV/K (12 min ultrasonication), respectively.

It has to be considered that different types of CNTs were applied in the reported former studies, whose structural and thus thermoelectric properties are most probably different. One may suppose that in SWCNTs with a higher carbon purity (compared to 75% carbon content of here used SWCNTs) the doping result in stronger effects. Additionally, different doping procedures, mostly in solution, were applied. For example, the studies by Piao et al. [21] used buckypapers prepared from SWCNTs from Thomas Swan & Co Ltd. and immersed them overnight in a PVP containing N-methyl-2-pyrrolidone (NMP) solution. In the study of Hata et al. [22], the polymer additives were dispersed with SWCNTs from Meijo Nano Carbon Co. Ltd. (Aichi, Japan) in NMP using ultrasonication, and the dispersion was then drop-casted on a polyimide substrate and dried at 60 °C (concentrations were not named). Ryan et al. [23] used an aqueous dispersion of MWCNTs (30 mg/mL) and PVP (120 mg/mL) sonicated before being coated two to seven times on vertically hanging yarns to achieve the n-type material. Nonoguchi et al. [24] performed the doping process by pouring 10 mL of a 5 wt% PVP solution in DMSO onto 5 mg of SWCNTs with very high carbon purity [37]. The mixture was sonicated and vacuum filtered to obtain n-type free-standing buckypapers. All these reported results have in common that the treatment of CNTs with PVP took place at room temperature. So, there was no temperature input during manufacture, in contrast to the melt blending process, which requires higher temperatures because the polymer has to be melted.

The main focus of our study was the preparation of melt-mixed polymer composites. It could be shown that PVP acts as an n-dopant for the SWCNTs in a polymeric matrix, at least to some extent. Negative Seebeck coefficients of up to −31 μV/K were obtained for PC composites, which is in accordance with the expectation based on the previous literature results. However, we found out that these negative values were not stable over time. After 1.5 years of sample storage under ambient conditions, the S-value of the composite without PVP was nearly reached again for all composites. It cannot be judged if this could also be the case for materials previously described in the literature, as such studies were not performed or not reported. For PBT as a polymer matrix, only a reduction of the S-values with increasing PVP content was measured. Here, too, the TE properties were not stable in the long term, so the S values increased with time. For PEEK composites, no n-doping effect of PVP was observed.

One reason for the different n-doping properties of the PVP in the composites can be seen in the high temperatures that have to be applied in the production of the composites based on the properties of the matrix polymers. PBT and PC are processed at 265 and 280 °C, respectively, which is in the range of the onset of the decomposition of PVP in the air. For PEEK processing at 360 °C, it has been shown that PVP has already been partially molecularly degraded (first maximum at 350 °C in the TGA study). This degradation will reduce the n-doping effect of PVP, especially in PEEK composites.

The difference in the effect of PVP in PBT and PC composites can be explained by the influence of the polymer matrix on the doping behavior of the SWCNTs themselves. For the SWCNT powder, an S-value of 39.6 ± 0.2 µV/K was measured [9]. In the composites with 2 wt% SWCNT, 37.8 ± 0.3 µV/K was determined in PC [10] and 59.4 ± 1.2 µV/K in PBT [9]. Thus, PBT shows a clear p-doping effect on SWCNTs compared to the rather small effect in PC. This means that in both composites, the PVP clearly shows an n-doping effect, but in the PBT composites, the initial value of the Seebeck coefficient is clearly higher than in those based on PC.

Unfortunately, the repetition of the measurements after 18 months for the PC and after 6 months for the PBT composites showed that the n-doping effect of the PVP was almost completely lost. Oxidative degradation of the PVP itself over time is suspected as the cause. The reason could be the high processing temperatures that are needed during the composite production by melt-mixing, which may have been an initialization for this later degradation step of PVP. Future trials could be carried out with polymer matrices having lower melting temperatures, such as poly(lactic acid) (PLA, melt processing temperature ca. 100 °C) or polycaprolactone (PCL, melt processing temperature ca. 120 °C). The strong hygroscopic of PVP could also play a role in the n-doping properties; although, this has not yet been investigated in depth. On a PC composite stored under vacuum at room temperature for 3 months, an increase in the Seebeck coefficient was measured that was comparable to that of the same composite after storage in the air (see Table S4). Since the electrical conductivities remained stable after storage, it is unlikely that a change in the SWCNT network structure occurred in the composites.

To overcome the problems with PVP, polyethylene glycol (PEG) is a suitable alternative additive for n-doping in polymer/SWCNT composites. It was shown that for PP [5] as well as PC, PBT, and PEEK [18] based composites with SWCNTs the addition of PEG leads to negative Seebeck coefficients. Unfortunately, only Luo et al. [5] investigated the long-term stability after 8 months of storage in air. It was found that the S-value increased from −44.6 to −26.4 µV/K, and the electrical conductivity remained constant (PP/0.8 wt% SWCNT + 5 wt% CuO + 4 wt% PEG). For other compositions with 8 and 10 wt% PEG (PP/2 wt% SWCNT + 5 wt% CuO), these trends were also found. For the PEG addition, instability of the TE properties over longer periods of time was also observed, which is, however, not quite as pronounced as was observed here for PVP. It is noteworthy that no change in electrical conductivity was measured for the composites containing PEG. This means that the changes during storage in air only affect the additive and not the electrically conductive CNT network.

## 5. Conclusions

PVP has been shown to have an n-doping effect on SWCNT buckypapers. However, it was very strongly dependent on the manufacturing conditions whether a lower positive (33.0 ± 1.7 µV/K) or a negative S-value (−8.1 ± 2.0 µV/K) could be achieved compared to 62.2 ± 0.6 µV/K for the untreated SWCNT buckypaper.

In melt-mixed composites, the polymer matrix, SWCNTs (in amounts of only 0.75 to 2.0 wt%), and PVP powder (1 to 10 wt%) were added together in the mixing compounder. Such fabrication method without the use of any solvents contributes to environmentally friendly approaches. It could be shown that a switch in the Seebeck coefficient is, in principle, possible, even if this could only be achieved for the polymer matrix PC. For PC/2 wt% SWCNT composite, the Seebeck coefficient switched from 37.8 ± 0.3 µV/K up to −31.5 ± 0.8 µV/K if 5 wt% PVP were added. For PBT as a matrix, a decrease in the Seebeck coefficient without reaching negative values could be found. A reduction of the Seebeck coefficient from 59.4 ± 1.2 µV/K (PBT/2 wt% SWCNT) up to 7.9 ± 0.8 µV/K could be reached with the addition of 8 wt% PVP. In PEEK/1 wt% SWCNT composites, the Seebeck coefficient value increased slightly from 48.0 ± 1.3 µV/K up to 54.3 ± 0.5 µV/K with a 3 wt% PVP content. On the other hand, the electrical volume conductivity of the composites increased (PC) or stayed nearly constant (PBT, PEEK) with PVP addition.

Unfortunately, long-term storage of the samples for 6–18 months illustrated that, despite the electrical conductivity, the achieved thermoelectric properties are not stable with time, and the switching effect was lost. Thus, in summary, PVP is, as a dopant additive, unsuitable for producing long-term stable melt-mixed composites with a negative Seebeck coefficient.

## Data Availability

The data presented in this study are available on request from the corresponding author.

## Supplementary Materials

The following supporting information can be downloaded at: https://www.mdpi.com/article/10.3390/mi14010181/s1, Table S1. TE properties of PC composites with SWCNT and PVP in different quantities (electrical volume conductivity, Seebeck coefficient, power factor, figure of merit ZT) measured at 40 °C; Table S2. TE properties of PBT composites with SWCNT and PVP in different quantities (Volume conductivity, Seebeck coefficient, power factor, figure of merit ZT) measured at 40 °C; Table S3. TE properties of PEEK composites with SWCNT and PVP in different quantities (electrical volume conductivity, Seebeck coefficient, power factor, figure of merit ZT) measured at 40 °C; Table S4. TE properties of PC composites with 2 wt% SWCNT and 15 wt% PVP (electrical volume conductivity, Seebeck coefficient, power factor, figure of merit ZT) measured at 40 °C. Composite prepared by laboratory scale extrusion by project partner AIMEN (Spain). Figure of merit ZT was calculated for all composites with the thermal conductivity of PC/1 wt% MWCNT of about 0.3 W/m·K [38].
