# Supplementary material for: Influence of Polyvinylpyrrolidone on Thermoelectric Properties of Melt-Mixed Polymer/Carbon Nanotube Composites
Source: Micromachines (Basel). 2023 Jan 11;14(1):181. doi: 10.3390/mi14010181 (PMC9863564; doi:10.3390/mi14010181)
Supplement: Supplementary file 1 [file micromachines-14-00181-s001.zip › micromachines-2144229-supplementary.pdf]

micromachines

Supporting information to

Influence of polyvinylpyrrolidone on thermoelectric properties of melt-mixed polymer/carbon nanotube composites

Beate Krause [1]*, Sarah Imhoff [1,2], Brigitte Voit [1,2], and Petra Pötschke [1]

[1] Leibniz-Institut für Polymerforschung Dresden e.V. (IPF), Hohe Str. 6, 01069 Dresden, Germany
[2] Chair Organic Chemistry of Polymers, Technische Universität Dresden, 01062, Dresden, Germany
• Correspondence: krause-beate@ipfdd.de; Tel.: +49 351 4658 736

Table S1. TE properties of PC composites with SWCNT and PVP in different quantities (electrical volume conductivity, Seebeck coefficient, power factor, figure of merit ZT) measured at 40°C

**Composite**	**Vol. conductivity σ (S/m)**	**Seebeck coeff. S (µV/K)**	**Power factor PF (µW/(m·K²))**	**ZT* (-)**
PC/ 0.75 wt% SWCNT [1]	0.9	39.5 ± 0.8	1.4 x 10^{-3}	1.5 x 10^{-6}
PC/ 0.75 wt% SWCNT + 1.5 wt% PVP	0.2 ± 0.0	14.0 ± 0.9	4.6 x 10^{-5}	4.8 x 10^{-8}
PC/ 1 wt% SWCNT [1]	1.0	36.7 ± 2.0	1.3 x 10^{-3}	1.4 x 10^{-6}
PC/ 1 wt% SWCNT + 2 wt% PVP	0.5 ± 0.0	9.1 ± 0.7	4.0 x 10^{-5}	4.2 x 10^{-8}
PC/ 2 wt% SWCNT [1]	1.0	37.8 ± 0.3	1.2 x 10^{-3}	1.5 x 10^{-6}
ʻʻ + 2 wt% PVP	1.9 ± 0.0	-19.6 ± 2.6	7.3 x 10^{-4}	7.7 x 10^{-7}
ʻʻ + 3 wt% PVP	2.3 ± 0.0	-30.6 ± 0.4	2.2 x 10^{-3}	2.3 x 10^{-6}
ʻʻ + 4 wt% PVP	2.0 ± 0.0	-30.0 ± 1.7	1.8 x 10^{-3}	1.9 x 10^{-6}
ʻʻ + 5 wt% PVP	2.7 ± 0.0	-31.5 ± 0.8	2.7 x 10^{-3}	2.9 x 10^{-6}
ʻʻ + 2 wt% PVP (after 18 month)	2.6 ± 0.3	27.1 ± 0.9	1.9 x 10^{-3}	2.0 x 10^{-6}
ʻʻ + 3 wt% PVP (after 18 month)	2.7 ± 0.1	24.0 ± 2.1	1.6 x 10^{-3}	1.6 x 10^{-6}
ʻʻ + 4 wt% PVP (after 18 month)	2.0 ± 0.1	23.1 ± 1.4	1.0 x 10^{-3}	1.1 x 10^{-6}
ʻʻ + 5 wt% PVP (after 18 month)	2.9 ± 0.2	22.4 ± 0.4	1.4 x 10^{-3}	1.5 x 10^{-6}

*) Figure of merit ZT was calculated for all composites with the thermal conductivity of PC/1 wt% MWCNT of 0.30 W/m·K [2]

Table S2. TE properties of PBT composites with SWCNT and PVP in different quantities (Volume conductivity, Seebeck coefficient, power factor, figure of merit ZT) measured at 40°C

Composite	Vol. conductivity σ (S/m)	Seebeck coeff. S (μV/K)	Power factor PF (μW/(m·K²))	ZT* (-)
PBT/ 2 wt% SWCNT [3]	22.0 ± 0.2	59.4 ± 1.2	7.6 x 10^{-2}	5.3 x 10^{-5}
ʹʹ + 2 wt% PVP	14.7 ± 7.6	25.0 ± 0.5	9.2 x 10^{-3}	6.4 x 10^{-6}
ʹʹ + 4 wt% PVP	8.2 ± 0.1	18.4 ± 0.7	2.8 x 10^{-3}	1.9 x 10^{-6}
ʹʹ + 6 wt% PVP	9.0 ± 5.0	11.2 ± 1.2	1.1 x 10^{-3}	7.7 x 10^{-7}
ʹʹ + 8 wt% PVP	8.3 ± 3.3	7.9 ± 0.8	5.2 x 10^{-4}	3.6 x 10^{-7}
ʹʹ + 10 wt% PVP	4.0 ± 0.0	18.3 ± 1.5	1.3 x 10^{-3}	9.2 x 10^{-7}
ʹʹ + 2 wt% PVP (after 6 month)	18.8 ± 9.8	52.4 ± 0.5	5.2 x 10^{-2}	3.6 x 10^{-5}
ʹʹ + 4 wt% PVP (after 6 month)	9.7 ± 0.4	47.6 ± 6.7	2.2 x 10^{-2}	1.5 x 10^{-5}
ʹʹ + 6 wt% PVP (after 6 month)	4.4 ± 0.0	48.1 ± 0.2	1.0 x 10^{-2}	7.0 x 10^{-6}
ʹʹ + 8 wt% PVP (after 6 month)	9.3 ± 3.8	47.4 ± 3.0	2.1 x 10^{-2}	1.4 x 10^{-5}
ʹʹ + 10 wt% PVP (after 6 month)	4.8 ± 0.1	49.6 ± 3.3	1.2 x 10^{-2}	8.2 x 10^{-6}

*) Figure of merit ZT was calculated for all composites with the thermal conductivity of PBT/2 wt% SWCNT Tuball of 0.46 W/m·K

Table S3. TE properties of PEEK composites with SWCNT and PVP in different quantities (electrical volume conductivity, Seebeck coefficient, power factor, figure of merit ZT) measured at 40°C

Composite	Vol. conductivity σ (S/m)	Seebeck coeff. S (μV/K)	Power factor PF (μW/(m·K²))	ZT* (-)
PEEK/ 1 wt% SWCNT [1]	6.2	48.0 ± 1.3	1.4 x 10^{-2}	1.1 x 10^{-5}
ʹʹ + 1 wt% PVP	12.4 ± 1.1	47.0 ± 1.9	2.7 x 10^{-2}	2.1 x 10^{-5}
ʹʹ + 2 wt% PVP	12.9 ± 0.8	49.7 ± 6.2	3.2 x 10^{-2}	2.5 x 10^{-5}
ʹʹ + 3 wt% PVP	12.8 ± 2.1	54.3 ± 0.5	3.8 x 10^{-2}	2.9 x 10^{-5}
ʹʹ + 1 wt% PVP (after 17 month)	13.7 ± 1.9	50.9 ± 0.8	3.5 x 10^{-2}	2.7 x 10^{-5}
ʹʹ + 2 wt% PVP (after 17 month)	14.2 ± 1.0	53.0 ± 3.6	4.0 x 10^{-2}	3.1 x 10^{-5}
ʹʹ + 3 wt% PVP (after 17 month)	16.9 ± 2.6	55.8 ± 1.7	5.3 x 10^{-2}	4.1 x 10^{-5}

*) Figure of merit ZT was calculated for all composites with the thermal conductivity of PEEK/1 wt% SWCNT Tuball of 0.40 W/m·K

Table S4. TE properties of PC composites with 2 wt% SWCNT and 15 wt% PVP (electrical volume conductivity, Seebeck coefficient, power factor, figure of merit ZT) measured at 40°C. Composite prepared by laboratory scale extrusion by project partner AIMEN (Spain)

Composite PC/2 wt% SWCNT+15 wt% PVP#	Vol. conductivity σ (S/m)	Seebeck coeff. S (μV/K)	Power factor PF (μW/(m·K²))	ZT* (-)
Direct after preparation (piece a)	12.7 ± 0.0	1.8 ± 0.2	4.1 x 10^{-5}	4.3 x 10^{-8}
after 3 month storage in air @25°C	12.0 ± 0.0	23.1 ± 0.1	6.4 x 10^{-3}	6.7 x 10^{-6}
Direct after preparation (piece b)	26.0 ± 0.1	0.7 ± 0.1	1.1 x 10^{-5}	1.2 x 10^{-8}
after 3 month storage in vacuum @25°C	26.3 ± 0.3	19.2 ± 0.1	9.6 x 10^{-3}	1.0 x 10^{-5}

#) Preparation using Thermo Scientific HAAKE PolyLab QC lab conical twin-screw extruder with three heating zones (temperature profile: 190°C – 220°C – 230°C; dye temperature 220°C; rotation speed 10-20 prm); premixing of components in a tubular: SWCNT and PVP for 15 min and then fillers with PC for 30 min

*) Figure of merit ZT was calculated for all composites with the thermal conductivity of PC/1 wt% MWCNT of about 0.3 W/m·K [2]

Determination of thermal conductivity:

The thermal conductivity of the composites was calculated from the product of thermal diffusivity, density, and specific heat capacity. The thermal diffusivity was measured on compression moulded round samples (diameter 12.3 mm, thickness 2 mm) through the plate thickness using the light flash apparatus LFA 447 NanoFlash (Netzsch-Gerätebau GmbH, Selb, Germany) at 25°C. The specific heat capacity of the composites was calculated by comparing the signal heights between the composite and the reference Pyroceram 9606 (with known specific heat capacity) using the LFA 447 NanoFlash. The density of composites was determined using a buoyancy method.

References

1. Konidakis, I.; Krause, B.; Park, G.-H.; Pulumati, N.; Reith, H.; Pötschke, P.; Stratakis, E. Probing the Carrier Dynamics of Polymer Composites with Single and Hybrid Carbon Nanotube Fillers for Improved Thermoelectric Performance. *ACS Applied Energy Materials* **2022**, *5*, 9770-9781, doi:10.1021/acsaem.2c01449.
2. Wegrzyn, M.; Ortega, A.; Benedito, A.; Gimenez, E. Thermal and electrical conductivity of melt mixed polycarbonate hybrid composites co-filled with multi-walled carbon nanotubes and graphene nanoplatelets. *J. Appl. Polym. Sci.* **2015**, *132*, doi:https://doi.org/10.1002/app.42536.
3. Krause, B.; Barbier, C.; Levente, J.; Klaus, M.; Pötschke, P. Screening of different carbon nanotubes in melt-mixed polymer composites with different polymer matrices for their thermoelectric properties. *Journal of Composites Science* **2019**, *3*, 106, doi:https://doi.org/10.3390/jcs3040106.
